# Effects of *Trichoderma harzianum* and *Bacillus subtilis* on the root and soil microbiomes of the soybean plant INTACTA RR2 PRO™

**DOI:** 10.3389/fpls.2024.1403160

**Published:** 2024-08-27

**Authors:** Everlon Cid Rigobelo, Luana Alves de Andrade, Carlos Henrique Barbosa Santos, Edvan Teciano Frezarin, Luziane Ramos Sales, Lucas Amoroso Lopes de Carvalho, Daniel Guariz Pinheiro, Daniel Nicodemo, Olubukola Oluranti Babalola, Maria Caroline Quecine Verdi, Mateus Mondin, Nicolas Desoignies

**Affiliations:** ^1^ Agricultural and Livestock Microbiology Postgraduate Program, São Paulo State University (UNESP), School of Agricultural and Veterinarian Sciences, Jaboticabal, São Paulo, Brazil; ^2^ Faculty of Agrarian and Veterinary Sciences, State University of São Paulo (UNESP), Jaboticabal, Brazil; ^3^ Food Security and Safety Focus Area, Faculty of Natural and Agricultural Sciences, North-West University, Mmabatho, South Africa; ^4^ University of São Paulo, College of Agriculture “Luiz de Queiroz”, Genetics Science Department, Piracicaba, Brazil; ^5^ Phytopathology, Microbial and Molecular Farming Lab, Center D’Etudes et Recherche Appliquée-Haute Ecole Provinciale du Hainaut Condorcet, Ath, Belgium

**Keywords:** *Bacillus subtilis*, microbiome, plant growth-promotion, soil microbiology, plant microbiology

## Abstract

**Introduction:**

Soybean is a significant export product for several countries, including the United States and Brazil. There are numerous varieties of soybean. Among them, a genetically modified type known as INTACTA RR2 PRO™ has been designed to demonstrate resistance to glyphosate and to produce toxins that are lethal to several species of caterpillars. Limited information is available on the use of *Trichoderma harzianum* and *Bacillus subtilis* to promote plant growth and their impact on the plant microbiome.

**Methods:**

This study aimed to evaluate the effects of these microorganisms on this soybean cultivar by analyzing parameters, such as root and shoot dry matter, nutritional status, and root and soil microbial diversity.

**Results:**

The results indicated that treatments with *B. subtilis* alone or in combination with *T. harzianum* as seed or seed and soil applications significantly enhanced plant height and biomass compared to the other treatments and the control. No significant differences in phosphorus and nitrogen concentrations were detected across treatments, although some treatments showed close correlations with these nutrients. Microbial inoculations slightly influenced the soil and root microbiomes, with significant beta diversity differences between soil and root environments, but had a limited overall impact on community composition.

**Discussion:**

The combined application of *B. subtilis* and *T. harzianum* particularly enhanced plant growth and promoted plant-associated microbial groups, such as Rhizobiaceae, optimizing plant-microbe interactions. Furthermore, the treatments resulted in a slight reduction in fungal richness and diversity.

## 1 Introduction

Soybean (*Glycine max*) is a crop produced by several countries and has a high economic value because it is the main crop responsible for producing oil and proteins for human consumption and animal production. Although genetic enhancement plays a significant role in the development of many crops, soybean plants have not experienced significant improvements in productivity (Liu et al., 2020). Using biotechnological tools, novel exogenous genes, such as *cry1* from *Bacillus thuringiensis*, which encode insecticidal proteins that enhance the use of transgenics, have been incorporated into soybean plants and serve as significant control methods with broad potential applications in agriculture. These environmentally friendly control measures have been implemented for various crops including soybean, corn, cotton, and rice (Gu et al., 2021). Among the products based on microorganisms, the main applications involve the biocontrol of pests and pathogens, especially in the case of filamentous fungi, in addition to biofertilization and growth promotion, which are more common among bacteria, especially plant growth-promoting rhizobacteria (dos Santos et al., 2020). With regard specifically to filamentous fungi, several studies have shown that their potential goes beyond biocontrol, with high potential for application in the field of biofertilization (Baron et al., 2018, 2019, 2020). However, there is a lack of studies confirming its potential *in planta*. Fungi can be classified as endophytic or mycorrhizal, based on their interactions with plants. Endophytic fungi typically reside within the tissues of aerial plant parts, such as stems and leaves, whereas mycorrhizal fungi exclusively inhabit plant roots. Both fungal groups are mutualistic and coexist with plants without causing any harm or negative effects (de Carvalho et al., 2020).

Endophytic and mycorrhizal fungi can act as plant growth promoters by increasing germination rate and improving seedling establishment, in addition to increasing plant resistance to biotic and abiotic stresses through the production of antimicrobial compounds, hormones, and other bioactive compounds (Baron et al., 2019; Chitnis et al., 2020). In addition, fungi can aid in the supply of nutrients to the soil, including macronutrients such as phosphorus, nitrogen, potassium, and magnesium or micronutrients such as zinc, iron, and copper (Khachatourians and Arora, 2001; Porras-Alfaro and Bayman, 2011; Balestrini et al., 2020).

*Trichoderma* spp. can promote plant growth through several mechanisms, such as stimulating the plant’s immune system and increasing plant resistance to pathogenic microorganisms by inducing systemic resistance. This can reduce the incidence and severity of the disease, resulting in healthier plants and higher yields. Moreover, *Trichoderma* can enhance the absorption of nutrients, particularly phosphorus and iron, which are frequently limiting factors for plant growth. On the other hand, the bacterium *B. subtilis* is also a plant growth-promoting bacterium with diverse abilities, such as biological nitrogen fixation (Sun et al., 2020), phosphorus solubilization (Ahmad et al., 2021), phytohormone production (Yarullina et al., 2020) and increased resistance to abiotic stresses (De Lima et al., 2022). Therefore, coinoculation of soybean plants with the fungus *T. harzianum* and *B. subtilis* may synergistically promote soybean growth.

Despite the recognition of the importance of fungi associated with plants, little is known about the effect of the application of the fungus *T. harzianum* alone or in combination with *B. subtilis* on the microbiome of the roots of the transgenic soybean variety INTACTA RR2 PRO™ and on plant growth-promoting parameters. Thus, this study was designed to investigate the beneficial effects of co-inoculation with *T. harzianum* and *B. subtilis* in two different application modes on the growth of soybean plants of the INTACTA RR2 PRO™ variety. Furthermore, this study aimed to evaluate the impact of these microbial applications on the composition and diversity of the plant’s native microbiota, providing insights into the potential improvements in crop productivity and microbial community dynamics.

## 2 Materials and methods

### 2.1 Microorganisms

The *B. subtilis* strain utilized in this study originated from the collection of the Agricultural Microbiology Laboratory at the UNESP campus in Jaboticabal, Brazil. This bacterium, initially isolated from a corn plant, was identified through sequencing, with its sequence available under GenBank accession number MZ133755. Concurrently, the *T. harzianum* fungus employed was isolated by Dr. Noemi Carla Baron Consentino during her doctoral research. This strain was sourced from soil on a rural property in Taquaritinga, São Paulo, Brazil. For inoculum development, *B. subtilis* was cultured in nutrient broth for 48 h at 28°C, whereas *T. harzianum* was cultured in potato dextrose broth for 14 days at 28°C. After the incubation period, the concentration was evaluated using the serial dilution method and standardized to 1 × 10^9^ CFU mL^-1^.

### 2.2 Experimental design of greenhouse experiments

The experimental setup was a completely randomized design within a greenhouse located in Jaboticabal, SP, Brazil (21° 15’ 17″ S and 48° 19’ 20″ W). This study aimed to evaluate the effects of microbial inoculations on the growth of soybean plants of variety INTACTA RR2 PRO™ using seven different treatments. These treatments included applying *T. harzianum* to seeds (T1), applying *B. subtilis* to seeds (T2), a combined application of both *T. harzianum* and *B. subtilis* to seeds (T3), application of *T. harzianum* to both seeds and soil (T4), application of *B. subtilis* to both seeds and soil (T5), and dual application of both microbes to seeds and soil (T6). The control group (T7) did not receive any microbial treatment. Each treatment was replicated across six pots, totaling 42 pots for the experiment.

The application via seed consisted of applying 200 mL of each or both microorganisms to 50 kg of seed, both at a concentration of 1 × 10^9^ colony forming unit (CFU) mL^-1^. For the treatments that received both microorganisms, there was a 20-minute drying interval between the application of the bacterium and the fungus. For application via soil, the inoculum at a concentration of 1 × 10^9^ CFU mL^-1^ was applied once directly to the soil in a volume of 10 mL per pot. In the treatments that received both microorganisms, each pot received 10 mL of each microorganism at the same volume and at the concentration mentioned above. During the 30-day study period, each treatment group received five weekly microbial re-inoculations through foliar applications, consisting of the same concentrations and volumes as the initial inoculation. Additionally, colonization of the roots by *T. harzianum* was confirmed through genetic transformation to incorporate the gene for Green Fluorescent Protein (*gfp*), enabling subsequent microscopic evaluation. The detailed methodology is provided in the **Supplementary Material**.

Three plants of the soybean variety INTACT RR2 PRO™ from Embrapa were initially planted in each 5-liter pot, which were subsequently thinned to two plants per pot after initial growth. The pots were filled up to 90% of their capacity with eutrophic red latosol soil, characterized by its chemical properties, including a pH of 6.9, 10% organic matter, 23 mg/dm³ available phosphorus, 0.7 mmolc/dm³ available potassium, 79 mmolc/dm³ calcium, 13 mmolc/dm³ magnesium, and 11 mmolc/dm³ hydrogen. Controlled environmental conditions were maintained in the greenhouse at a temperature of 24 ± 2°C, 50 ± 2% relative humidity, and a light cycle of 16:8 hours of light to dark, fitting the region’s Aw climate classification by Köppen and Geiger. In this experiment, soybean plants were assessed for several growth parameters. The plant height was measured from the apex to the base of the plant. The biomass was then processed by splitting the shoots from the roots, which were each dried in a forced ventilation oven at 65°C for 72 to 96 h, and subsequently weighed on a semi-analytical scale. The total dry mass of the plants was calculated by summing the weights of dried shoots and roots.

### 2.3 Determination of nitrogen and phosphorus in shoots and roots

Five hundred micrograms of dried and ground plant samples were weighed and placed into 50 mL digestion tubes, which were left to decouple at room temperature for 1.5 hours. The tubes were then positioned in a digestion block, initially heated to 80°C for 20 min before the temperature was increased to 160°C. The tubes were monitored and removed once the material ascended the tube walls, and most of the HNO_3_ evaporated, leaving a clear solution. After cooling, 1.3 mL the concentrated HClO_4_ was added to each tube. The tubes were returned to the block, the temperature was increased to 210°C, and digestion was deemed complete when the solution turned colorless with dense white vapor of HClO_4_ and H_2_O formed above the dissolved material. The tubes were cooled and the contents were diluted to 25 mL with water in a snap-cap glass (Wan et al., 2020).

For phosphorus analysis, 1 mL of the digested sample was transferred to a test tube, to which 4 mL of water and 2 mL of reagent mix (comprising equal parts of 5% ammonium molybdate and 0.25% vanadate) were added. The mixture was allowed to rest for 15 min before measuring the absorbance at 420 nm using a UV-VIS spectrophotometer (Meier et al., 2020).

### 2.4 DNA extraction, and sequencing of microbiomes from roots and soil

Roots were manually harvested using surgical gloves and immediately stored in sterilized plastic containers. To dislodge the rhizospheric soil, roots were placed in a 50 mL conical tube containing 35 mL of phosphate buffer and 0.02% Tween 20 and vortexed for 2 min. The roots were then transferred to sterile paper towels using sterilized forceps and subsequently transferred to 50 mL centrifuge tubes for superficial sterilization. The sterilization involved treating the plant tissues were sterilized with 100% ethanol for 3 min, 2% sodium hypochlorite for 2 min, and 70% ethanol for 3 min, based on a modified protocol from Cao et al. (2005). To confirm the sterilization efficacy, the final wash was cultured on nutrient agar plates and checked for the absence of microbial growth. Following sterilization, the roots were pooled by treatment and macerated using a sterile mortar and pestle in liquid nitrogen. Genomic DNA was extracted from these samples using the PowerMax Soil DNA Extraction Kit (Mo Bio Laboratories, Carlsbad, CA, USA), according to the manufacturer’s specifications. DNA concentration and purity were assessed using fluorometry (Qubit™ 3.0, Invitrogen) and spectrophotometry (NanoDrop™ 1000, Thermo Fisher Scientific), respectively, to determine the A260/A280 ratio.

For bacterial community analysis, the V4 region of the 16S rRNA gene was amplified using primers 515F (5′-GTGCCAGCMGCCGCGGTAA-3′) and 806R (5′-GGACTACHVGGGTWTCTAAT-3′), with modifications (Caporaso et al., 2012; de Souza et al., 2016). For the fungal community was targeted using the primers ITS3 (5′-GCATCGATGAAGAACGCAGC-3′) and ITS4 (5′-TCCTCCGCTTATTGATATGC-3′). PCR amplification was conducted using the HotStarTaq Plus Master Mix Kit (Qiagen) over 30 cycles with specific thermal cycling conditions (94°C for 3 min, followed by 28 cycles of 94°C for 30 s, 53°C for 40 s, and 72°C for 1 min, and a final elongation step at 72°C for 5 min). Additionally, PNA clamp sequences were incorporated to prevent amplification of mitochondrial and ribosomal 16S rRNA sequences. The PCR amplicons were sequenced using an Illumina MiSeq platform. The raw data can be found in the NCBI Sequence Read Archive (SRA) database under the BioProject PRJNA1141482.

### 2.5 Bioinformatics analysis

Quality assessment of the sequenced data was conducted using the FastQC software (v.0.11.9) (Andrews, 2010). The identification and localization of sequencing primers and barcodes were achieved through the “search_oligodb” function of USEARCH (v.11.0.667) (Edgar, 2010), and removed using Atropos (v.1.1.31) (Didion et al., 2017). To enhance data integrity, Fastp (v. 0.23.2) (Chen et al., 2018) was utilized to discard sequences with an average Phred quality score below Q25, applying the parameter “-average_qual 25.” Given the paired-end layout of sequencing, reads were merged using PEAR (version 0.9.11) (Zhang et al., 2014).

The quality-assured reads were then subjected to the DADA2 pipeline (Callahan et al., 2016), starting with the “filterAndTrim” function set to an expected error limit of two (“maxEE = 2”). This was followed by the “learnErrors” function to estimate error probabilities. Based on these probabilities, sequences were corrected using the “dada” function, which facilitates the identification of Amplicon Sequence Variants (ASVs). For taxonomic classification, bacterial ASVs were aligned against the SILVA database (v.138.1) (Quast et al., 2012), whereas fungal ASVs were compared with the UNITE database (v.7.0) (Abarenkov et al., 2010). ASVs not classified within the expected kingdoms (Bacteria or Fungi) or identified as potential contaminants, including chloroplast and mitochondrial sequences, were excluded from the analysis to ensure the accuracy and relevance of the microbial community profiles obtained. ITS samples from treatments T1, T5, T6, and T7 were excluded from further analyses because of the lack of usable sequences.

The diversity within and between microbial communities was analyzed to understand their ecological variance. Alpha diversity was assessed using the “alpha” function of the R package “microbiome” (v.1.16.0) (Lahti and Shetty, 2012), which calculated the observed richness along with Shannon and Simpson diversity indices. Beta diversity was determined by calculating Bray-Curtis dissimilarities using the “distance” function of the R package “phyloseq” (McMurdie and Holmes, 2013). This analysis helped to understand the compositional diversity and differences across the samples. Graphical representations of both relative abundance and diversity metrics were generated using “ggplot2” (v.3.3.6) (Wickham, 2011) in R (v.4.1.2) (R Core Team, 2023).

### 2.6 Statistical analysis

Statistical analyses were conducted to evaluate the effect of different treatments on plant growth parameters and their nitrogen and phosphorus contents. Analysis of variance (ANOVA) was performed using the F-test within the AgroEstat program (Barbosa and Maldonado Junior, 2015). Where significant differences were detected, mean comparisons were carried out using the Scott-Knott test at a 5% probability level. Furthermore, to associate the plant growth attributes and their nitrogen and phosphorus contents with the treatments, a Principal Component Analysis (PCA) was performed on the standardized values. This analysis was performed using the “FactoMineR” package (Lê et al., 2008) in the R program.

Diversity within microbial communities, inferred through alpha diversity measures, was statistically evaluated using Two-way ANOVA (inoculation and application mode). Differences between the environments (soil and root) were also evaluated. These analyses were performed using the R package “agricolae” (v.1.3.5) (Mendiburu, 2023), with the significance level set at p < 0.05. For beta diversity, differences between the microbial communities were assessed using PERMANOVA (function “adonis” of the R package “vegan”; v.2.6.2) (Oksanen et al., 2019), with a p-value of < 0.05, which was statistically significant. To further explore the relationships and patterns within the community data, Principal Coordinates Analysis (PCoA) was employed to reduce the multidimensionality of the distance metrics. The results from the PCoA are subsequently visualized in a graph.

## 3 Results

### 3.1 Microbial inoculation effects on plant growth and nutrient content

**Figure 1:**
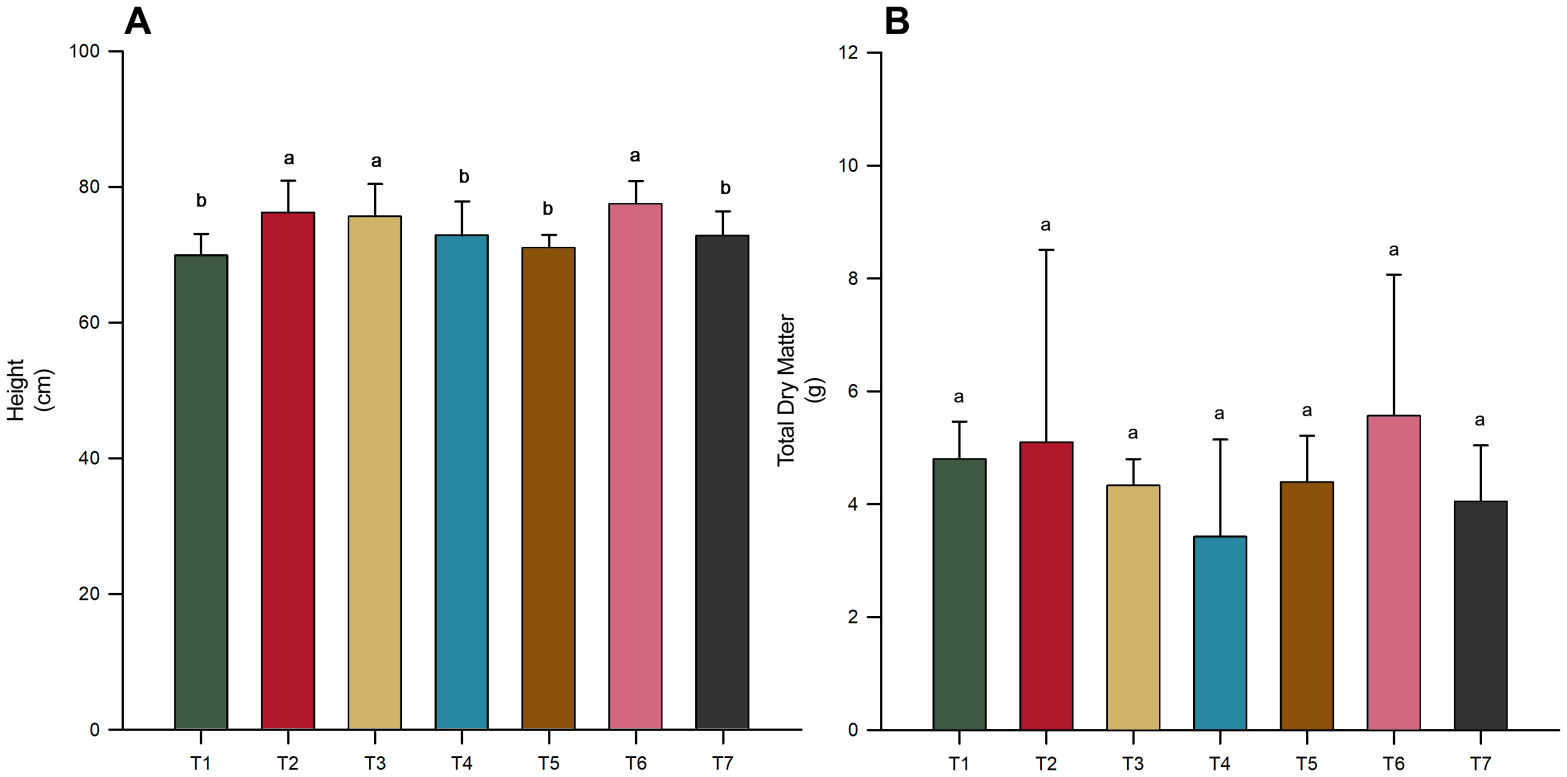
Impact of microbial inoculations on soybean variety INTACTA RR2 PRO™ growth. **(A)** Plant height of the soybean, measured in centimeters. Treatments include T1 (*Trichoderma harzianum*), T2 (*Bacillus subtilis*), and T3 (co-inoculation of *T. harzianum* and *B. subtilis*) applied to seeds; T4, T5, and T6 represent the same treatments applied to both seeds and soil, with T7 as the control. **(B)** Total dry matter of soybean plants, measured in grams, under the same treatment conditions. Statistically significant differences between treatments are indicated by different letters, as determined by the Scott–Knott test at the 5% probability level.

In this study, was investigated the effects of microbial inoculation on the growth of the soybean variety INTACTA RR2 PRO™, focusing on variations in the response to different treatments. These treatments included the application of *T. harzianum*, *B. subtilis*, and their co-inoculation, either directly to seeds or combined with soil application. **Figure 1** illustrates the effect of these treatments on soybean growth, presenting data on plant height (**Figure 1A**) and total dry matter (**Figure 1B**). The results indicated that plants treated with *B. subtilis* either alone (T2) or in combination with *T. harzianum* (T3 and T6) as a seed and soil application exhibited significantly greater heights compared to other treatments (**Figure 1A**) (*p*-value < 0.05). Notably, these treatments differed significantly from the control (T7), which had lower height. Compared to these treatments (T2, T3, and T6), those with solo inoculation of *T. harzianum* (T1 and T4) also showed lower performance, but did not differ from the control (T7). Despite these differences, with greater heights in some treatments, no significant differences were noted in relation to the total dry matter (**Figure 1B**).

**Figure 2:**
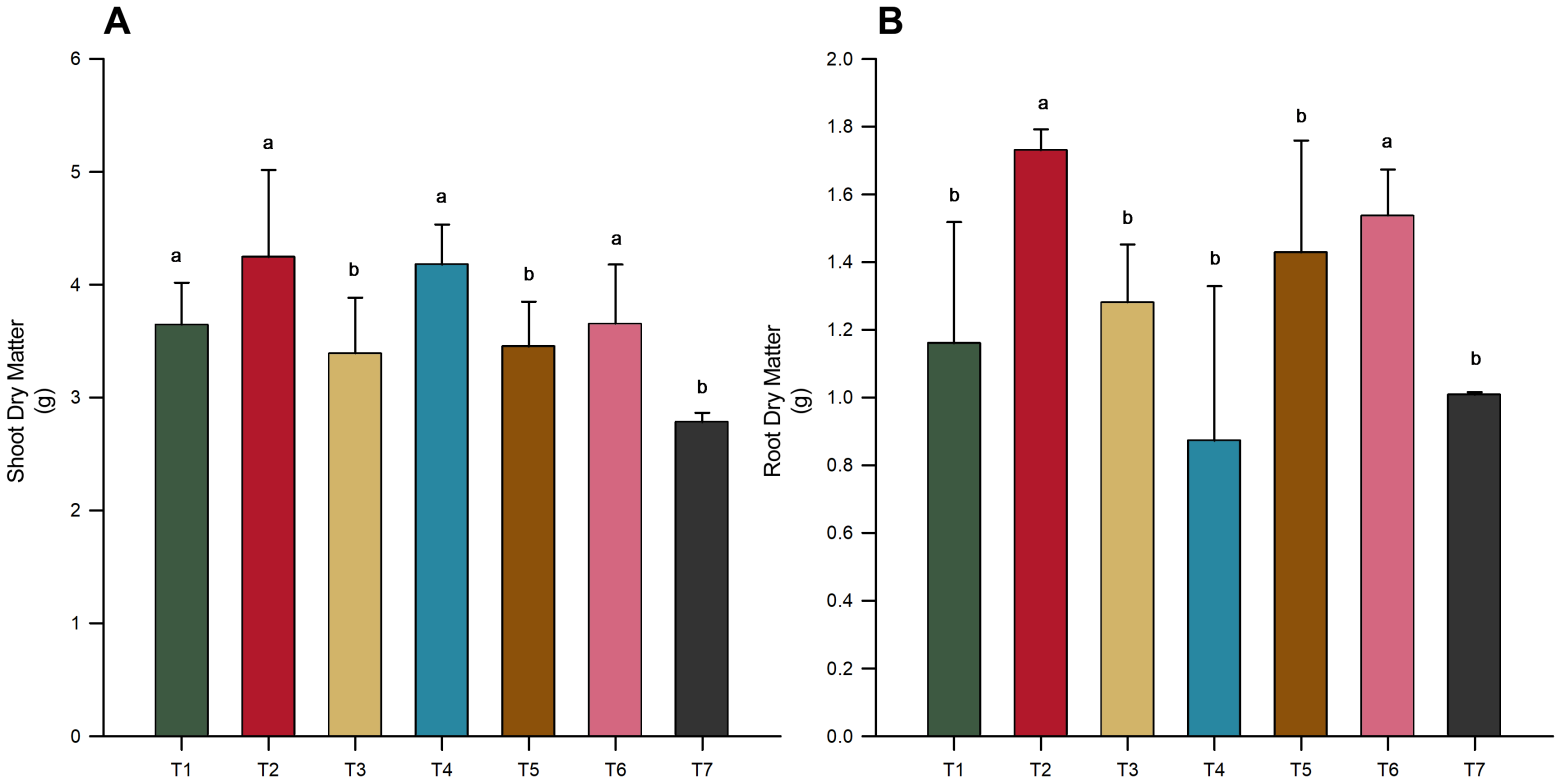
Impact of microbial inoculations on soybean variety INTACTA RR2 PRO™ biomass. **(A)** Shoot Dry Matter of the plants, expressed in grams. Treatments include T1 (*Trichoderma harzianum*), T2 (*Bacillus subtilis*), and T3 (co-inoculation of *T. harzianum* and *B. subtilis*) applied to seeds; T4, T5, and T6 represent the same treatments applied to both seeds and soil, with T7 as the control. **(B)** Root Dry Matter of the same soybean variety, measured in grams, following the identical treatment regimen. Significant differences between treatment results, as indicated by different letters, are established by the Scott–Knott test at the 5% probability level.

Despite the lack of significant differences in total dry matter across treatments (**Figure 1B**). Significant variations were observed in both shoot and root biomass when treatments were analyzed independently (**Figure 2**), revealing a more nuanced impact of microbial inoculation on plant growth dynamics (*p*-value < 0.05). Notably, treatment T2 (*B. subtilis* applied to seeds) resulted in the highest shoot (**Figure 2A**) and root dry matter (**Figure 2B**), significantly outperforming the control (T7) and other treatments. Similarly, the co-inoculation treatment (T6) of *T. harzianum* and *B. subtilis*, applied to both seeds and soil, was effective in increasing shoot and root biomass (**Figure 2**). Furthermore, inoculation with *T. harzianum* in both application modes (T1 and T4) was also effective in increasing shoot biomass, but there was no increase in root biomass (**Figure 2**).

**Figure 3:**
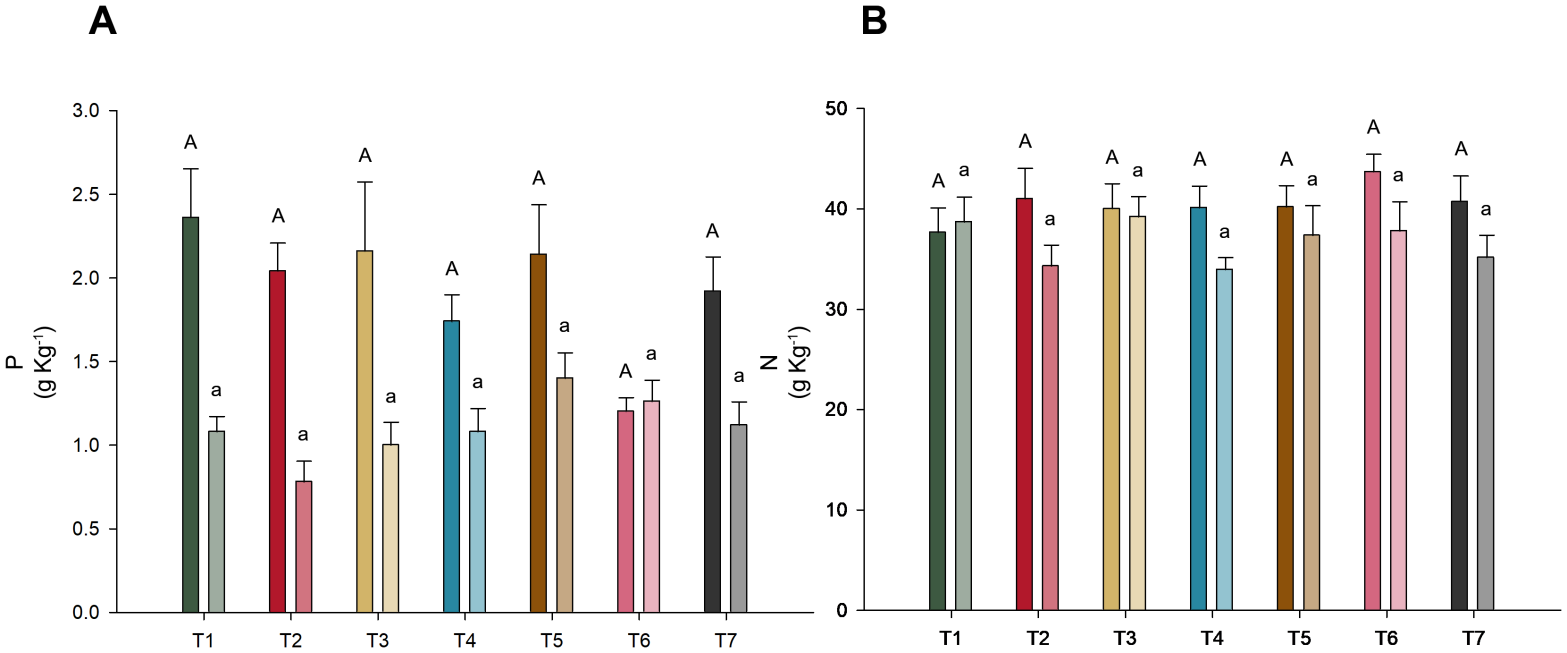
Nutrient concentration in soybean variety INTACTA RR2 PRO™ plants subject to microbial inoculations. **(A)** Concentration of phosphorus in the soybean plants, measured in grams per kilogram. Treatments include T1 (*Trichoderma harzianum*), T2 (*Bacillus subtilis*), and T3 (co-inoculation of *T. harzianum* and *B. subtilis*) applied to seeds; T4, T5, and T6 represent the same treatments applied to both seeds and soil, with T7 as the control. **(B)** Concentration of nitrogen phosphorus in the shoots and roots of the same soybean variety, expressed in grams per kilogram. Dark-colored bars indicate shoot concentrations, while light-colored bars denote root concentrations. Statistical significance between treatment effects within shoots (denoted by capital letters) and roots (denoted by lowercase letters) is indicated by different letters, as determined by the Scott–Knott test at the 5% probability level.

In the assessment of phosphorus and nitrogen concentrations across different microbial treatments, no significant differences were detected (*p*-value < 0.05), as shown in **Figure 3**. However, it is noteworthy that the phosphorus levels in the shoots of T6 (*T. harzianum* + *B. subtilis* applied to seeds and soil) were notably lower than those in the other treatments.

**Figure 4:**
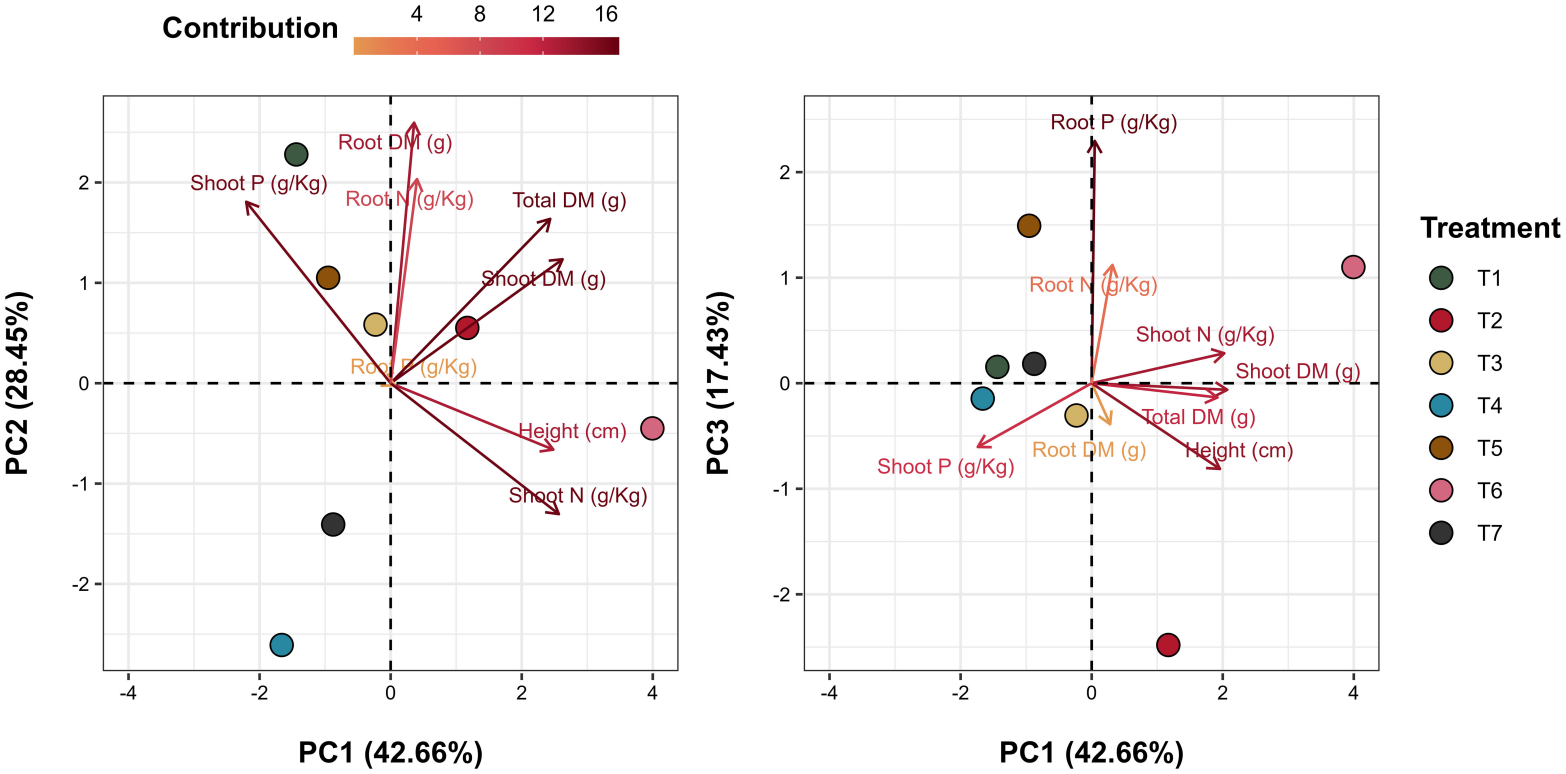
Principal Component Analysis (PCA) biplot of plant growth and nutritional content. Vectors illustrate the plant growth and nutritional content variables, and points represent the treated samples. The graph on the left depicts the first (PC1) and second (PC2) principal components, while the graph on the right displays the interaction between the first (PC1) and third (PC3) principal components. Together, these three principal components account for 88.54% of the total variance explained in the samples. Treatments include T1 (*Trichoderma harzianum*), T2 (*Bacillus subtilis*), and T3 (co-inoculation of *T. harzianum* and *B. subtilis*) applied to seeds; T4, T5, and T6 represent the same treatments applied to both seeds and soil, with T7 as the control.

Principal Component Analysis (PCA) effectively visualized the effects of microbial treatments on plant growth and nutrient content in INTACTA RR2 PRO™ soybean plants, with the biplots of the first three principal components capturing 88.54% of the total variance (**Figure 4**). The PCA biplot for PC1 and PC2 highlighted strong correlations between root dry matter and root nitrogen content, as well as between plant height and nitrogen content in the shoots. In the biplot contrasting PC1 and PC3, a significant linkage was observed between root phosphorus and shoot nitrogen. Treatment-specific inspection revealed distinct effects in T1 (*T. harzianum* applied to seeds), T2 (*B. subtilis* applied to seeds), and T6 (*T. harzianum* + *B. subtilis* applied to seeds and soil). Each treatment demonstrated unique vector orientations in the PCA plots, reflecting their specific influence on the growth parameters and nutrient dynamics of soybean plants. This was noted by the close association between T1 and shoot phosphorus content (**Figure 4**, PC1 vs. PC2), T2 with root dry matter (**Figure 4**, PC1 vs. PC3), and T6 with shoot height and nitrogen content (**Figure 4**, PC1 vs. PC2).

### 3.2 Microbial inoculation effects on root and soil microbiome

To explore the effects of microbial inoculation on the microbiome associated with the soil and roots of INTACTA RR2 PRO™ soybean plants, amplicon sequencing targeting the V4 16S rRNA regions for bacteria and the ITS regions for fungi was employed. This approach yielded 399,574 reads for the bacterial communities and 413,473 reads for the fungal communities. However, the quality control process revealed significant variability in data usability. Notably, post-quality control, the soil ITS dataset from treatments T1, T5, T6, and T7 resulted in no usable reads.

To ensure a focus on well-identified microbial communities, further filtering was applied to retain only amplicon sequence variants (ASVs) that could be classified at least at the phylum level. Moreover, the adequacy of sequencing depth was confirmed through rarefaction analysis. The stabilization of the rarefaction curves for both the bacterial and fungal datasets, as illustrated in **Supplementary Figure S1A, B**, indicates that the sequencing effort was sufficient to capture the diversity present.

To assess the diversity of microbial communities was evaluated the alpha diversity measures of the observed richness of ASVs and Shannon and Gini-Simpson indices. In the roots, the highest nominal richness was observed in treatment T1 (*T. harzianum* applied to seeds) (**Table 1**). In contrast, for soil samples, treatment T2 (*B. subtilis* applied to seeds) exhibited the highest richness and diversity (both Shannon and Gini-Simpson) in soil samples. Despite this, T2 showed a lower Shannon diversity index in the roots, surpassing only the control treatment (T7). For the fungal communities, the highest richness and diversity indices in the roots were observed in the control treatment (T7). However, the lack of complete data on soil fungal communities prevents a complete comparison in this environment.

**Table 1:** Alpha diversity metrics for bacterial and fungal communities in soil and root.

Treatment		Richness		Shannon		Gini-Simpson	
		Root	Soil	Root	Soil	Root	Soil
Bacteria	T1	73	29	3.17	2.92	0.93	0.93
	T2	40	159	2.88	4.47	0.91	0.98
	T3	57	51	3.33	3.40	0.95	0.95
	T4	45	57	3.17	2.30	0.94	0.77
	T5	61	29	3.11	3.00	0.92	0.93
	T6	42	12	3.19	2.28	0.94	0.88
	T7	40	37	2.83	3.18	0.90	0.94
Fungi	T1	7	–	1.48	–	0.73	–
	T2	10	19	1.76	2.52	0.77	0.90
	T3	4	19	0.93	2.28	0.56	0.82
	T4	3	22	0.62	2.24	0.33	0.83
	T5	19	–	2.23	–	0.84	–
	T6	16	–	2.28	–	0.85	–
	T7	22	–	2.65	–	0.91	–

**Figure 5:**
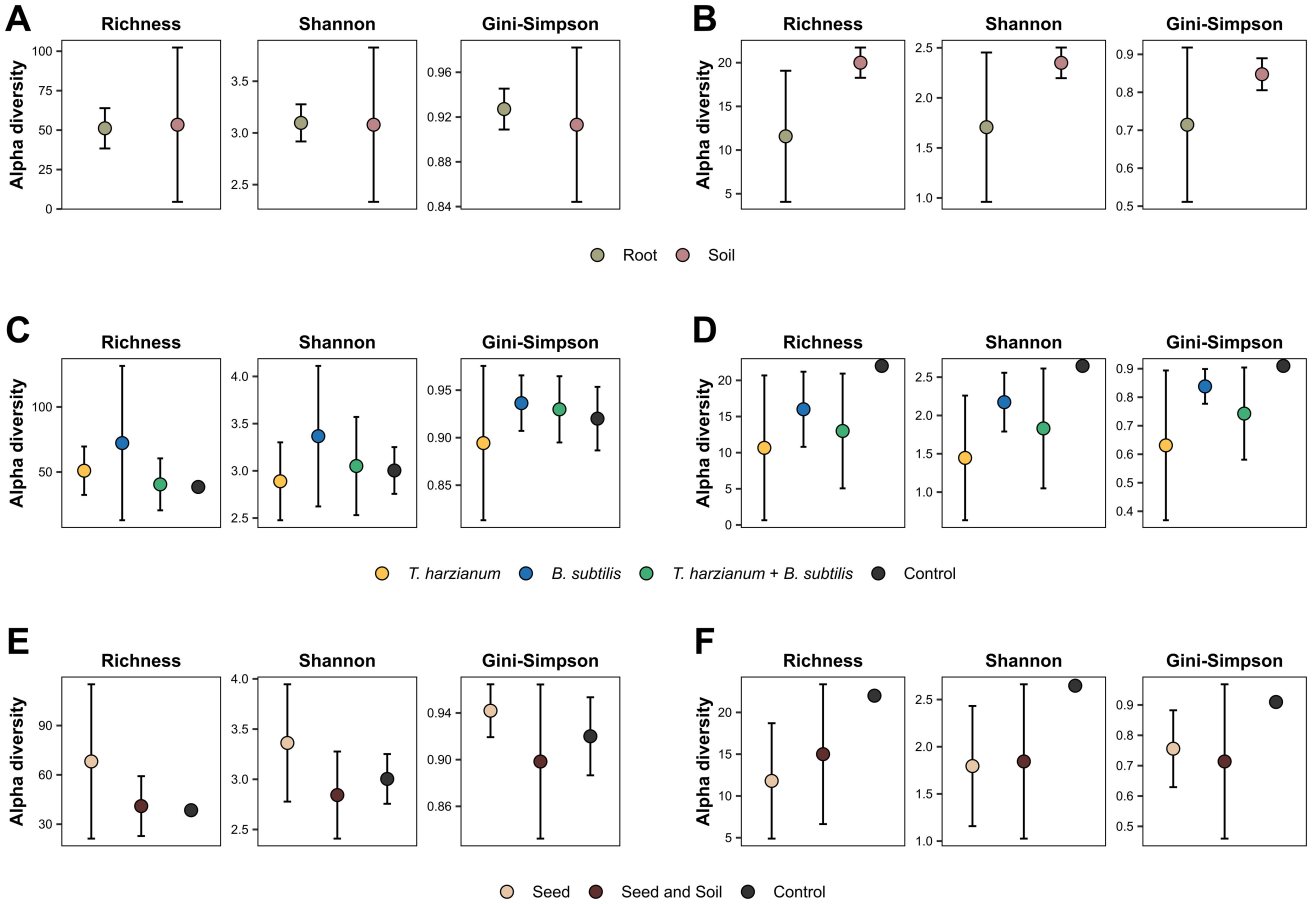
Alpha diversity measures of the microbial communities across different factors. The graphs illustrate the average Richness, Shannon and Gini-Simpson diversity indices for bacterial **(A, C, E)** and fungal **(B, D, F)** communities. The indices are analyzed and presented from the perspective of different experimental factors. **(A, B)** Diversity measures segmented by the origin environment of the samples (soil vs. roots). **(C, D)** Diversity indices based on the type of inoculant used in the treatment. **(E, F)** Measures of diversity according to the method of application (seed vs. seed and soil). No significant differences were found across the groups (ANOVA test, *p*-value > 0.05).

The diversity indices were also compared in relation to the environment of origin of the samples (root vs. soil), the treatment factors (inoculation and application method), and their interaction (Two-way ANOVA). There was no statistically significant effect for any comparison in either dataset (p < 0.05). It is noteworthy that there was great dispersion of the data (**Figure 5**) and overlap of the distribution range of the means, thus explaining the lack of statistical differences.

**Figure 6:**
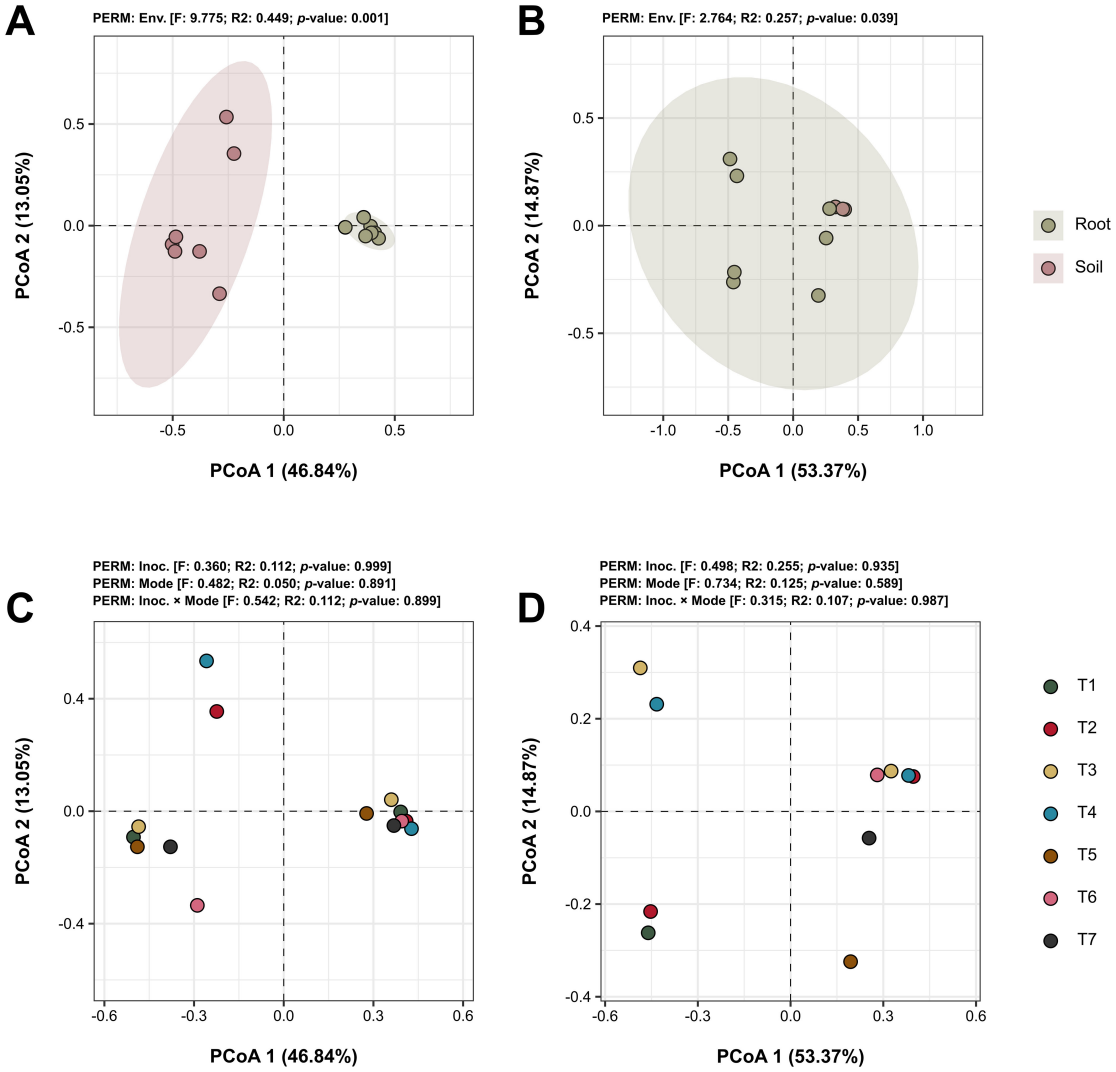
Beta diversity analyses of the microbial communities across different factors. Principal Coordinate Analysis (PCoA) plots based on Bray-Curtis distances, illustrating the beta diversity within bacterial **(A, C)** and fungal **(B, D)** communities across different treatment samples. Graphs A and B depict the comparison between source environments of the samples (roots vs. soil), while C and D explore the diversity differences between treatment factors (type of inoculant and application method) and their interactions (treatments). Statistical values are provided above each graph. Treatments include T1 (*Trichoderma harzianum*), T2 (*Bacillus subtilis*), and T3 (co-inoculation of *T. harzianum* and *B. subtilis*) applied to seeds; T4, T5, and T6 represent the same treatments applied to both seeds and soil, with T7 as the control.

Beta diversity, which assesses the variation in microbial community composition between samples, was evaluated in this study using Bray-Curtis distances to compare the impacts of various experimental factors. The analysis focused on comparing microbial community compositions relative to the environment of origin (root vs. soil) and different microbial treatments applied (treatments). Significant differences in microbial community composition were observed between the soil and root environments. For bacteria, beta diversity showed differences with a p-value of 0.001 (**Figure 6A**), indicating distinct bacterial communities in soil compared to roots. Fungi also showed significant differences, although less pronounced, with a p-value of 0.039 (**Figure 6B**). When assessing the impact of treatment factors, including both isolated and interacting effects of microbial inoculations, no significant differences were detected in the community compositions of either bacteria (**Figure 6C**) or fungi (**Figure 6D**), with all p-values exceeding 0.05.

When exploring the compositional profile of the bacterial data, was observed a distinct dominance of specific taxa that varied significantly between treatments. In the root samples, the phylum Proteobacteria was predominant, ranging from 75 to 90% across treatments, followed by Actinobacteria, which ranged from 8 to 20%, and Bacteroidetes. In contrast, soil samples predominantly featured Proteobacteria (50–75%), followed by the phylum Firmicutes, which was absent in the root samples. Treatment T1 (*T. harzianum* seed application) exhibited a higher abundance of Proteobacteria than the other treatments, whereas treatment T6 (*T. harzianum* + *B. subtilis* applied to seeds and soil) displayed a greater diversity of phyla (**Supplementary Figure S2A**; **Supplementary Table 1**: “%Phylum”).

Within the root samples, Gammaproteobacteria accounted for 13–70%, followed by Alphaproteobacteria, which constituted 25–30%. The highest percentage of Gammaproteobacteria was found in treatment T1 (*T. harzianum* seed application), whereas treatment T5 (*B. subtilis* seed and soil application) promoted the greatest abundance of Alphaproteobacteria. Generally, the bacterial diversity was higher in the soil samples than in the root samples. In the soil, Gammaproteobacteria ranged from 15 to 50%, and Alphaproteobacteria ranged from 50 to 52%. Treatment T2 (*B. subtilis* seed application) showed the greatest diversity of bacterial classes (**Supplementary Figure S2B**; **Supplementary Table 1**: “%Class”). In the roots, the order Enterobacterales ranged from 3 to 30%, followed by Burkholderiales, which ranged from 2 to 25%. The highest relative abundance of the order Rhizobiales was observed in treatment T5 (*B. subtilis* seed and soil application) (**Supplementary Table 1**: “%Order”). The soil samples showed similar trends, especially for the T7 control treatment, which exhibited high bacterial diversity [**Supplementary Figure S2C**; **Supplementary Table 1**: “%Order”)].

**Figure 7:**
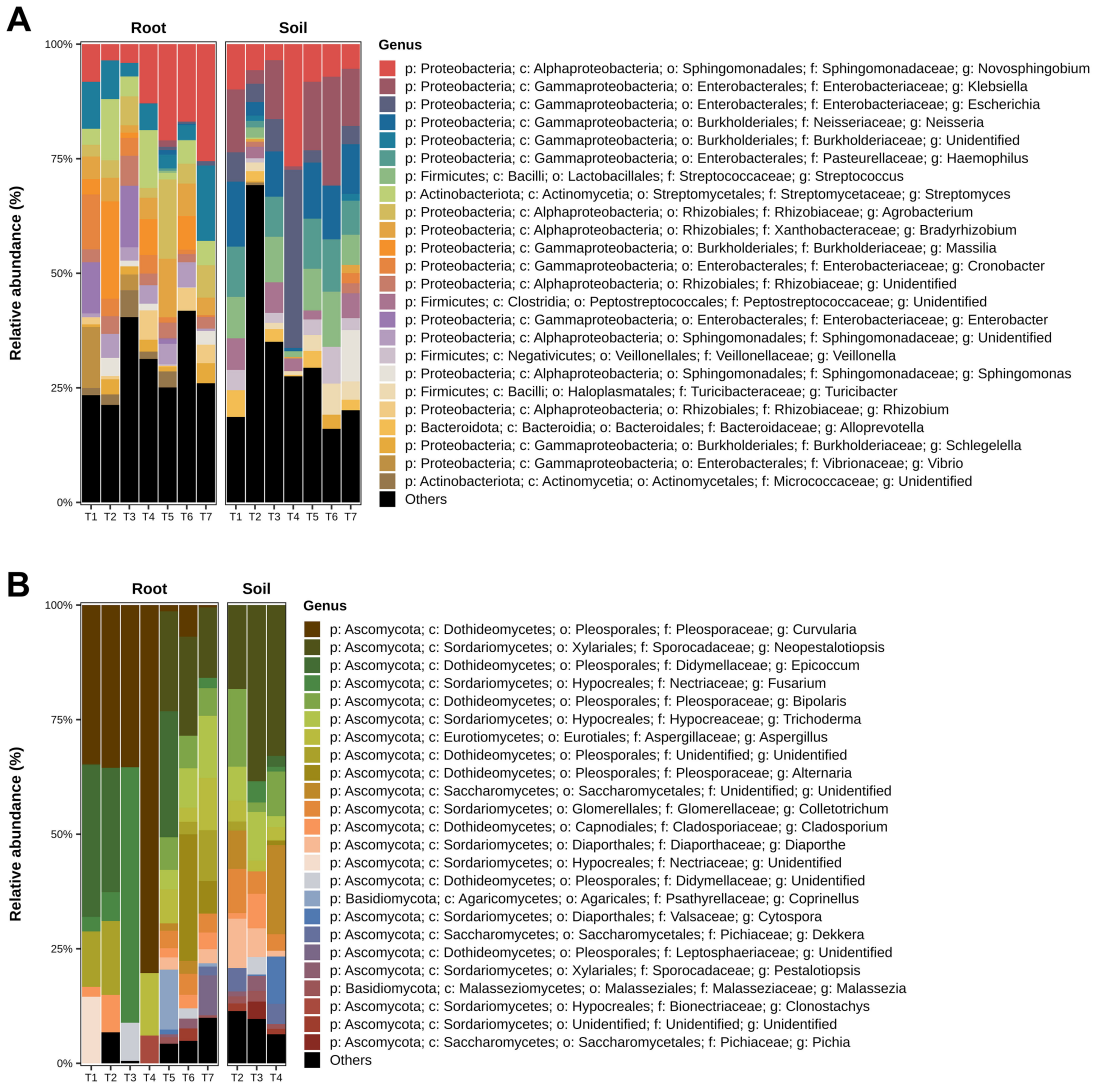
Relative Abundance of the most prevalent bacterial **(A)** and fungal **(B)** genera in the samples, differentiated between roots and soil environments. The graphs illustrate the proportions of each genus, with those of lower abundance grouped and labeled as “Others.”. Treatments include T1 (*Trichoderma harzianum*), T2 (*Bacillus subtilis*), and T3 (co-inoculation of *T. harzianum* and *B. subtilis*) applied to seeds; T4, T5, and T6 represent the same treatments applied to both seeds and soil, with T7 as the control.

The family Enterobacteriaceae was predominant in the root samples, followed by Sphingomonadaceae and Bulkholderiaceae (**Supplementary Figure S2D**; **Supplementary Table 1**: “%Family”). Treatment T2 (*B. subtilis* seed application) showed greater diversity in the soil samples than the other treatments. In the roots, the relative abundance of bacterial genera did not vary significantly between treatments, with the highest prevalence observed for the genera *Novosphingobium*, *Klebsiella*, *Escherichia*, *Neisseria*, *Haemophilus*, *Streptococcus*, *Streptomyces*, *Agrobacterium*, and *Bradyrhizobium*. Soil samples demonstrated a greater prevalence of *Novosphingobium*, *Klebsiella*, *Escherichia*, *Neisseria*, *Haemophilus*, and *Streptococcus*, and a lower prevalence of *Bradyrhizobium*, *Massila*, and *Cronobacter* compared to root samples (**Figure 7A** **;** **Supplementary Table 1**: “%Genus”).

When analyzing the compositional profile of fungal data at the phylum taxonomic level, results free from unclassified sequences revealed a dominance of the phylum *Ascomycota*, followed by *Basidiomycota* (**Supplementary Figure S3A** **;** **Supplementary Table 2**: “%Phylum”). Notably, *Basidiomycota* were more abundant in treatment T5 (*B. subtilis* seed and soil application). At the class level, *Dothideomycetes* was predominant, ranging from 45 to 75% across the treatments, with *Sordariomycetes* at 7–55% and *Eurotiomycetes* at 5–12% (**Supplementary Figure S3B** **;** **Supplementary Table 2**: “%Class”). Treatment T1 (*T. harzianum* seed application) displayed the lowest diversity of fungal classes, whereas treatment T6 (*T. harzianum* + *B. subtilis* seed and soil application) showed the highest diversity of fungal phyla, indicating a significant impact of co-inoculation on fungal diversity. In the soil samples, while relative abundances were generally similar across treatments, treatment T3 (*T. harzianum* + *B. subtilis* seed application) exhibited a higher diversity of fungal classes (**Supplementary Figure S3B**; **Supplementary Table 2**: “%Class”).

In both the root and soil samples, the fungal orders Pleosporales, Xylariales, Hypocreales, and Saccharomyces were prevalent. Notably, treatment T7 (control) in the soil samples exhibited the greatest diversity of orders (**Supplementary Figure S3C** **;** **Supplementary Table 2**: “%Order”). Across the samples, the most common fungal families were Pleosporaceae, Sporocadaceae, Didymeliaceae, and Aspergilaceae (**Supplementary Figure S3D** **;** **Supplementary Table 2**: “%Family”). In the root samples, the control treatment (T7) and treatment T5 (*B. subtilis* seed application) exhibited a high diversity of fungal families (**Supplementary Figure S3D**; **Supplementary Table 2**: “%Family ”). In the soil samples, the treatment that promoted the greatest diversity of fungal families was T2 (*B. subtilis* seed application) (**Supplementary Figure S3D** **;** **Supplementary Table 2**: “%Family “).

The genus *Curvularia* was the most abundant in the root samples, followed by *Neopestalotiopsis*, *Epicoccum*, *Fusarium*, *Bipolaris*, and *Trichoderma*. In contrast, *Neopestalotiopsis* was the most prevalent genus in the soil samples, followed by *Epicoccum* and *Fusarium*. The control treatment (T7) exhibited the greatest diversity of fungal genera (**Figure 7B**; **Supplementary Table 2**: “%Genus”).

## 4 Discussion

This study investigated the effects of microbial inoculation on the growth and nutrient content of INTACTA RR2 PRO™ soybean plants. The results indicated that plants treated with *B. subtilis* alone (T2) or in combination with *T. harzianum* as seed (T3) or seed and soil (T6) exhibited significantly greater heights than other treatments (**Figure 1A**). Treatments T2 and T6 also significantly outperformed the control (T7) in terms of shoot and root biomass (**Figure 2**). This suggests that *B. subtilis* has a robust effect on promoting soybean growth, possibly because of its known benefits in enhancing nutrient uptake and stimulating plant growth hormones. *B. subtilis* has been widely recognized as an effective plant growth-promoting rhizobacterium (PGPR) for various crops, including soybean, because of its ability to solubilize phosphate, produce indole acetic acid, and induce systemic resistance (Hashem et al., 2019; Chagas Junior et al., 2022). Recent studies have shown that multiple strains of *B. subtilis* can significantly enhance soybean growth and yield. For instance, field trials with *B. subtilis* Bs10 resulted in up to a 31.8% increase in soybean biomass and yield compared to controls (Braga Junior et al., 2021), and greenhouse experiments have shown improvements in agronomic characteristics, such as shoot and root biomass, nodulation, and nutrient accumulation (Chagas Junior et al., 2022). Additionally, *B. subtilis* UFMT-Pant001 strain was found to positively affect plant height, biomass, and various growth parameters across different soybean cultivars (Marco et al., 2023).

Our findings are also consistent with those of recent studies demonstrating the synergistic effects of the studied microorganisms as plant growth-promoting agents. When inoculated together, *B. subtilis* and *T. harzianum* enhance plant growth parameters such as height, leaf area, and biomass, while reducing the need for chemical fertilizers (Silva et al., 2023). Furthermore, this microbial consortium has the potential to induce systemic resistance in plants, thereby enhancing their defense against pathogens (Chowdappa et al., 2013). For instance, co-inoculation of *B. subtilis* and *T. harzianum* effectively controls chocolate spot disease, reducing the efficacy of chemical fungicides (Firdu et al., 2022). Additionally, biofilms produced using *Trichoderma* and *B. subtilis* exhibit superior plant growth-promoting traits, such as increased antifungal activity and the production of ammonia, indole acetic acid, and siderophores (Triveni et al., 2013).

In terms of nutrient content, no significant differences were detected in phosphorus and nitrogen concentrations across the treatments (**Figure 3**). However, it is noteworthy that some treatments had close correlations with these variables, such as treatments T1 (*T. harzianum* applied to seeds) and T6 (*T. harzianum* + *B. subtilis* seed and soil application), which, in addition to obtaining the highest nominal averages (**Figure 3**), were closely associated with phosphorus and nitrogen content in the shoots, respectively (**Figure 4**). This is also supported by the literature, since *Trichoderma* species are known to be capable of producing organic acids and solubilizing phosphorus (Gaind, 2016; Li et al., 2015; Bononi et al., 2020) and the combination of *T. harzianum* and *B. subtilis* can also improve soil fertility by increasing nutrient availability (Silva et al., 2023).

Furthermore, our results revealed interesting patterns regarding the method of microbial inoculation. Specifically, the *B. subtilis* treatment performed better when applied to seeds only (T2) compared to the seed and soil application (T5) in terms of plant height and biomass (**Figures 1A**, **2**). For other treatments, there were no notable differences between application methods, except for shoot dry matter, where the combination of *T. harzianum* and *B. subtilis* applied to seeds and soil (T6) was superior to seed-only application (T3) (**Figure 2A**). These findings suggest that the mode of inoculation plays a crucial role in optimizing the benefits of microbial treatment. Although studies specifically addressing application modes for the microorganisms in this study are limited, previous research on rhizobia application in soybeans has shown that soil application resulted in higher soybean yields than seed application (Martyniuk et al., 2018). Conversely, research on legumes has indicated that seed application slightly improves nodulation, grain yield, and nitrogen fixation compared with liquid in-furrow application (Denton et al., 2017). With these mixed responses, both in the present work and in the literature, the effectiveness of the application method appears to be specific to each inoculant or combination, and it is difficult to generalize beyond the conditions studied here.

In addition to evaluating vegetative parameters, was assessed the effects of treatments on soil and root microbiomes associated with INTACTA RR2 PRO™ soybean plants. The values observed in the richness and diversity measures exhibited distinct patterns among treatments (**Table 1**; **Figure 5**). However, statistical analysis did not reveal any significant differences based on the sample source environment (soil or root) or the factors evaluated in the treatments (inoculants and their application methods). This finding is in line with previous studies indicating that, although inoculation can influence indigenous microbial communities in the short term, these changes are typically transient (Trabelsi and Mhamdi, 2013). For example, *B. subtilis* PTS-394 showed only a brief impact on the rhizosphere microbiota of tomatoes, lasting up to 14 days for eukaryotes and 3 days for bacteria (Qiao et al., 2017). Similarly, periodic inoculations with phosphate-solubilizing and N2-fixing bacteria initially affected the bacterial community composition, but the community eventually exhibited resilience to subsequent inoculations (Wang et al., 2021).

Despite the lack of statistically significant differences, the fungal microbiome of the control treatment (T7) displayed the highest index values for richness and diversity (**Table 1**; **Figure 5**). This could be attributed to the absence of inoculants, which may allow native fungal communities to thrive without external competitive pressure. Alternatively, the high diversity observed in the control could suggest that the inoculants exert a controlling effect on certain fungal organisms, thereby reducing the overall diversity by suppressing specific fungal taxa. This is consistent with numerous studies that have reported the multifaceted capabilities of *B. subtilis* (Mardanova et al., 2017; Hashem et al., 2019; Kiesewalter et al., 2021) and *T. harzianum* (Braun et al., 2018; Swathy et al., 2024) in suppressing pathogenic fungi, potentially leading to the reduced fungal diversity observed in treated samples.

Our beta diversity analysis revealed significant differences in microbial community composition between the soil and root environments (**Figures 6A, B**). For bacterial communities, Bray-Curtis distances indicated highly distinct compositions between the soil and roots, with a p-value of 0.001 (**Figure 6A**). This suggests that bacterial communities are more specialized and vary depending on the environment of origin. Similarly, fungal communities also displayed significant differences between soil and root environments, although these differences were less pronounced (**Figure 6B**). Despite these environmental differences, when assessing the impact of treatment factors, including both isolated and interacting effects of the microbial inoculations, no significant differences were detected in the community composition of either bacteria (**Figure 6C**) or fungi (**Figure 6D**). This lack of significant variation suggests that, while microbial inoculations can influence specific microbial groups, their overall impact on the broader community composition may be limited. These findings highlight the inherent resilience and stability of the native microbial communities in the soybean rhizosphere, which may quickly re-establish their original structure following inoculation interventions, as discussed for alpha diversity measures (Trabelsi and Mhamdi, 2013; Qiao et al., 2017; Wang et al., 2021).

When assessing the influence of the treatments on specific microbial groups, significant variations in taxonomic composition were noted. Predominant groups in the bacterial and fungal communities were well conserved at broader taxonomic ranks, such as Phylum and Class (**Supplementary Figures S2**, **S3**; **Supplementary Tables 1**, **2**). However, at more specific ranks, such as Order, Family, and Genus, wide fluctuations in relative abundance were observed, even among the most abundant taxa. Additionally, the diversity of lower abundance groups often collapsed as “Others” in taxonomic profile views, suggesting a large latent diversity in certain treatments. This was particularly notable in treatment T2 (*B. subtilis* as seed application), where a broad range of bacterial taxa was maintained across various taxonomic levels, underscoring the impact of treatment on promoting microbial diversity (**Supplementary Figure S2**; **Supplementary Table 1**).

In the bacterial community, a considerable increase in the order Rhizobiales and its family Rhizobiaceae was observed in treatments T5 (*B. subtilis*) and T6 (*T. harzianum* + *B. subtilis* seed and soil application), both involving inoculation in the seed and soil. The Rhizobiaceae family includes diverse plant-associated bacteria that play crucial roles in plant-microbe interactions. In the present study, was observed three agriculturally relevant genera of Rhizobiaceae among the most abundant: *Agrobacterium* and rhizobia *Bradyrhizobium* and *Rhizobium* (**Figure 7A**). Although the genus *Agrobacterium* is widely recognized for its ability to transfer DNA into plant cells and induce tumor formation (Nester, 2015), rhizobial species are known for their symbiotic association with leguminous plants, forming root nodules where they fix atmospheric nitrogen (Rodríguez-Navarro et al., 2011; Nakei et al., 2022). This increase highlights the potential of these treatments to enhance the presence of beneficial nitrogen-fixing bacteria, which could contribute to improved plant health and growth.

At the genus level, notable shifts were observed between the soil and root environments. Soil samples showed greater abundance of genera such as *Klebsiella*, *Neisseria*, and *Haemophilus*, whereas root samples had higher abundance of *Streptomyces*, *Agrobacterium*, and *Bradyrhizobium*. These shifts suggest that different microbial communities have adapted to specific niches within the soybean rhizosphere. Plants can modulate their microbiome through root exudates, whereas bacteria have evolved specific mechanisms to colonize this niche (Jacoby et al., 2017; Roux et al., 2023). For fungal communities, the control treatment (T7) exhibited greater evenness among the most abundant genera, implying a higher fungal diversity in the absence of inoculants. This could indicate that the application of microbial treatments selectively suppresses certain fungal taxa, thereby altering the overall community structure and reducing the diversity of the treated samples.

## 5 Conclusion

Our study demonstrated that microbial inoculation with *B. subtilis* and *T. harzianum* significantly enhanced the growth and biomass of INTACTA RR2 PRO™ soybean plants. The mode of inoculation plays a crucial role, with seed-only and combined seed and soil applications showing different effects on plant growth parameters. Although no significant differences in nutrient content were observed, some treatments were closely correlated with phosphorus and nitrogen levels, indicating their potential role in nutrient dynamics. Microbial inoculations had a transient effect on soil and root microbiomes, with significant differences in beta diversity between soil and root environments, but limited impact on overall community composition due to the resilience of native microbial communities. The observed shifts in specific microbial groups, such as the increase in Rhizobiaceae, underscore the potential of these treatments to optimize plant-microbe interactions. These findings highlight the importance of optimizing microbial inoculation strategies for sustainable agriculture, warranting further research to explore their long-term impacts and validate their efficacy under diverse conditions.

## Data Availability

The datasets presented in this study can be found in online repositories. The names of the repository/repositories and accession number(s) can be found in the article/**Supplementary Material**.

## Supplementary material

Supplementary Figure 1 Rarefaction curves of bacterial **(A)** and fungal **(B)** amplicons, each showing the number of Amplicon Sequence Variants (ASVs) as a function of sequencing depth. The curves demonstrate the progression towards sufficient sequencing coverage to capture the ASV diversity in the samples.

Supplementary Figure 2 Relative Abundance of the most prevalent bacterial phyla **(A)**, classes **(B)**, orders **(C)** and families **(D)** in the samples, differentiated between roots and soil environments. The graphs illustrate the proportions of each bacterial taxon, with those of lower abundance grouped and labeled as “Others.”. Treatments include T1 (*Trichoderma harzianum*), T2 (*Bacillus subtilis*), and T3 (co-inoculation of *T. harzianum* and *B. subtilis*) applied to seeds; T4, T5, and T6 represent the same treatments applied to both seeds and soil, with T7 as the control.

Supplementary Figure 3 Relative Abundance of the most prevalent fungal phyla **(A)**, classes **(B)**, orders **(C)** and families **(D)** in the samples, differentiated between roots and soil environments. The graphs illustrate the proportions of each fungal taxon, with those of lower abundance grouped and labeled as “Others.”. Treatments include T1 (*Trichoderma harzianum*), T2 (*Bacillus subtilis*), and T3 (co-inoculation of *T. harzianum* and *B. subtilis*) applied to seeds; T4, T5, and T6 represent the same treatments applied to both seeds and soil, with T7 as the control.
