# Supplementary material for: Effects of Trichoderma harzianum and Bacillus subtilis on the root and soil microbiomes of the soybean plant INTACTA RR2 PRO™
Source: Front Plant Sci. 2024 Aug 27;15:1403160. doi: 10.3389/fpls.2024.1403160 (PMC11383790; doi:10.3389/fpls.2024.1403160)
Supplement: Supplementary file 4 [file Table1.docx]

**Effects of *Trichoderma harzianum* and *Bacillus subtilis* on the root and soil microbiomes of the soybean plant INTACTA RR2 PRO™**

Everlon Cid Rigobelo ^1*^, Luana Alves de Andrade ^1^, Carlos Henrique Barbosa Santos ^1^, Edvan Teciano Frezarin ^1^, Luziane Ramos Sales ^1^, Lucas Amoroso Lopes de Carvalho ^1^, Daniel Guariz Pinheiro ^1^, Daniel Nicodemo ^2^, Olubukola Oluranti Babalola ^3^, Maria Carolina Quecine Verdi ^4^, Mateus Mondin ^4^, Nicolas Desoignies ^4^

^1^ Agricultural and Livestock Microbiology Postgraduate Program, São Paulo State University (UNESP), School of Agricultural and Veterinarian Sciences, Jaboticabal, São Paulo, Brazil

^2^ Faculty of Agrarian and Veterinary Sciences, State University of São Paulo (UNESP), Jaboticabal, Brazil

^3^ Food Security and Safety Niche Area, Faculty of Natural and Agricultural Sciences, North‒West University, Mmabatho 2735, South Africa

^4^ University of São Paulo, College of Agriculture “Luiz de Queiroz”, Genetics Science Department, Piracicaba, Brazil

^5^ Phytopathology, Microbial and Molecular Farming Lab, Center D'Etudes et Recherche Appliquée-Haute Ecole Provinciale du Hainaut Condorcet, Ath, Belgium.

***** Correspondence:[everlon.cid@unesp.br](mailto:everlon.cid@unesp.br)

ORCID: 0000-0002-9734-3338

**Supplementary Material**

**Transformation of *Trichoderma harzianum* mediated by *Agrobacterium tumefaciens***

To evaluate *T. harzianum* colonization in soybean roots, we performed the transformation of fungal lineages mediated by *Agrobacterium tumefaciens* to introduce specific markers. For this, we used a disarmed *A. tumefaciens* strain EHA05 containing the plasmids pFAT-GFP (Montoya et al., 2021) and pCAM-DsRed (Villena et al., 2020), provided by Prof. Dr. Maria Caroline Quecine Verdi from the University of São Paulo (ESALQ/USP). The pFAT-GFP vector includes the hygromycin B resistance gene (*hph*) driven by the glyceraldehyde-3-phosphate dehydrogenase (*gpd*) promoter from *Glomerella cingulata* and the green fluorescent protein (*gfp*) gene from *Aequorea victoria*. The pCAM-DsRed vector contains the *hph* gene driven by the *gpd* promoter from *Aspergillus nidulans* and the red fluorescent protein gene (*DsRed*) from *Discosoma* sp.

The transformed *A. tumefaciens* EHA05 cells were cultured in YEP media (10 g yeast extract, 10 g peptone, 5 g NaCl, 15 g agar, and 1000 mL distilled water; pH 7.0) with spectinomycin and rifampicin (100 μg/mL each). Prior to transformation, fungal strains were assessed for hygromycin B susceptibility by cultivating them on PDA for 7 days and then transferring 8 mm discs to PDA plates with various hygromycin B concentrations (0, 5, 10, 25, 50, 100, 200, 400 μg/mL) at 26°C for 7 days. The concentration that completely inhibited growth was chosen for transformation.

For transformation, the *A. tumefaciens* culture with binary vectors was incubated in YEP media (25 mL) with 300 μg/mL spectinomycin and 100 μg/mL rifampicin for 24 h at 26°C ± 2°C under 200 rpm agitation (Bernardi-Wenzel et al., 2016). The culture was diluted to an OD of 0.2 at 660 nm in induction medium (MI) containing 10 mM K_2_HPO_4_, 10 mM KH_2_PO_4_, 2.5 mM NaCl, 2 mM MgSO_4_, 0.7 mM CaCl_2_, 9 μM FeSO_4_, 4 mM NH_4_SO_4_, 10 mM glucose, 0.5% glycerol (pH 5.3), 40 mM MES, and 200 μM acetosyringone. The mixture was incubated under the same conditions for 6 h or until reaching an OD of 0.6 at 660 nm. At the target OD, the bacterial culture was mixed with a conidial suspension (10^6^ conidia/mL) of each fungus at a 1:1 ratio. Aliquots of 200 μL were applied to filter paper (8 μm, J. Prolab, Brazil) and/or nylon membranes (0.45 μm Amersham Hybon N+ or GE Healthcare) on MI plates (with 1.5% agar) with 200 μM acetosyringone. Plates were incubated at 25°C ± 2°C for 96 h, then transferred to PDA plates with the selected hygromycin B concentration and 300 μg/mL cefoxitin sodium to eliminate bacterial cells. Transformant growth was observed after 7 to 20 days at 28°C. Transformants were analyzed using fluorescence microscopy. Mycelia were cultured on PDA with hygromycin B, and after 4–7 days at 28°C, coverslips were mounted on slides with sterile distilled water and sealed for microscopic analysis using 480 nm filters for GFP and 545 nm filters for *DsRed*.

Transformation was further confirmed by colony PCR to amplify the *gfp*, *DsRed*, and *hph* genes using specific primers: glGFP5 and glGFP3 (Fitzgerald et al., 2003) for *gfp*, U61 and U62 (Eckert et al., 2005) for *DsRed*, and hph1 and hph2 (Ghadmagahi et al., 2022) for *hph*. PCR reactions were conducted in 25 μL volumes with 0.2 mM dNTPs, 3.7 mM MgCl2, 1× buffer (50 mM KCl; 20 mM Tris-HCl; pH 8.4), Taq DNA polymerase (0.05 U/μL), 0.2 μM primers, and template DNA. The program was 94°C for 5 min; 30 cycles of 94°C for 1 min, 60°C for 1 min, 72°C for 1 min; and a final extension at 72°C for 7 min, followed by analysis on a 1.2% agarose gel. The mitotic stability of transformants was tested by subculturing for five generations on PDA without hygromycin B from monosporic cultures grown on PDA with the antibiotic. Stability was confirmed by regrowth on PDA with hygromycin B after five transfers. After the plant experiment, root material was collected and taken to the Epigenetics Laboratory of the Genetics Department of ESALQ/USP for imaging to verify colonization, as shown in the figure below.

**Supplementary Mat. Figure 1**. Colonization of soybean roots by GFP-modified *Trichoderma harzianum*.


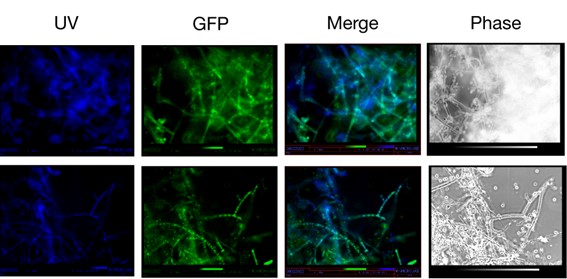


**References**

Montoya, M. R. A., Massa, G. A., Colabelli, M. N., and Ridao, A. del C. (2021). Efficient *Agrobacterium tumefaciens*-mediated transformation system of *Diaporthe caulivora*. *J Microbiol Methods* 184. doi: 10.1016/j.mimet.2021.106197

Villena, C. I. F., Gomes, R. R., Fernandes, L., Florencio, C. S., Bombassaro, A., Grisolia, M. E., et al. (2020). *Agrobacterium tumefaciens*-mediated transformation of *Fonsecaea monophora* and *Fonsecaea erecta* for host-environment interaction studies. *Journal of Fungi* 6, 1–11. doi: 10.3390/jof6040325

Bernardi-Wenzel, J., Quecine, M. C., Azevedo, J. L., & Pamphile, J. A. (2016). Agrobacterium-mediated transformation of Fusarium proliferatum. *Genet Mol Res*, *15*(2), 01-12.

Fitzgerald, A. M., Mudge, A. M., Gleave, A. P., & Plummer, K. M. (2003). Agrobacterium and PEG-mediated transformation of the phytopathogen Venturia inaequalis. *Mycological research*, *107*(7), 803-810.

Eckert, M., Maguire, K., Urban, M., Foster, S., Fitt, B., Lucas, J., & Hammond-Kosack, K. (2005). Agrobacterium tumefaciens-mediated transformation of Leptosphaeria spp. and Oculimacula spp. with the reef coral gene DsRed and the jellyfish gene gfp. *FEMS microbiology letters*, *253*(1), 67-74.

Ghadmagahi, V., Zafari, D., and Soltani J. (2022). *Agrobacterium tumefaciens*-mediated transformation of *Trichoderma viridescens*. *Mycol Iran* 9, 67–73. doi: 10.22043/MI.2022.360018.1230
